# Crystal structure of 16-ferrocenylmethyl-3β-hydroxy­estra-1,3,5(10)-trien-17-one: a potential chemotherapeutic drug

**DOI:** 10.1107/S2056989016008446

**Published:** 2016-05-27

**Authors:** José A. Carmona-Negrón, Mariola M. Flores-Rivera, Zaibeth Díaz-Reyes, Curtis E. Moore, Arnold L. Rheigold, Enrique Meléndez

**Affiliations:** a University of Puerto Rico, Department of Chemistry, PO Box 9019, Mayaguez, PR 00681, Puerto Rico; b University of Puerto Rico, Department of Chemical Engineering, PO Box 9000, Mayaguez, PR 00681, Puerto Rico; c University of California–San Diego, Department of Chemistry and Biochemistry, Urey Hall 5128, 9500 Gilman Drive, La Jolla, CA 92093-0358, USA

**Keywords:** crystal structure, anti­cancer compound, ferrocene, medicinal chemistry

## Abstract

A new ferrocene complex, 16-ferrocenylmethyl-3β-hy­droxy­estra-1,3,5(10)-trien-17-one dimethyl sulfoxide monosolvate, [Fe(C_5_H_5_)(C_24_H_27_O_2_)]·C_2_H_6_OS, has been synthesized and structurally characterized by single-crystal X-ray diffraction techniques. The mol­ecule crystallizes in the space group *P*2_1_ with one mol­ecule of dimethyl sulfoxide. A hydrogen bond links the phenol group and the dimethyl sulfoxide O atom, with an O⋯O distance of 2.655 (5) Å. The ferrocene group is positioned in the β face of the estrone moiety, with an O—C—C—C torsion angle of 44.1 (5)°, and the carbonyl bond length of the hormone moiety is 1.216 (5) Å, typical of a C=O double bond. The average Fe—C bond length of the substituted Cp ring [Fe—C(Cp*)] is similar to that of the unsubstituted one [Fe—C(Cp)], *i.e.* 2.048 (3) *versus* 2.040 (12) Å. The structure of the complex is compared with those of estrone and eth­oxy­methyl­estrone.

## Abstract 2

A new ferrocene complex, 16-ferrocenylmethyl-3β-hy­droxy­estra-1,3,5(10)-trien-17-one, has been synthesized and structurally characterized by single-crystal X-ray diffraction techniques. The ferrocenylmethyl group is positioned at the β face of the estrone moiety; as a result, a new stereogenic center is formed leading to an *R* stereochemical configuration. No head-to-tail hydrogen bonding is observed in the crystal packing, as is the case in estrone and other derivatives.

## Chemical context   

The discovery of cisplatin anti­neoplastic activity was a notable event in medicinal chemistry history, opening new alternatives and routes on the use of metal-based drugs and their structure–activity relationships in cancer chemotherapy. However, its remarkable success (Galanski *et al.*, 2005 ▸; Sandler *et al.*, 2011 ▸) came at the high cost of undesired detrimental side effects (neurotoxicity, nephrotoxicity, *etc*; Pabla & Dong, 2008 ▸). In this context, our research group has been working on other transition metals (*e.g*., titanium, iron, vanadium and tungsten, among others) with promising results for chemotherapeutic applications (Domínguez-García *et al.*, 2013 ▸; Ramos *et al.*, 2014 ▸; Vera *et al.*, 2014 ▸). Recently, particular attention has been focused on the anti­neoplastic activity of ferrocene complexes (Richard *et al.*, 2015 ▸) due to their desired physical and chemical properties such as aqueous stability and high synthetic homology to benzene chemistry, with the advantage that they exhibit fewer toxic side effects than cisplatin. Our group has been working on the synthesis and application of ferrocene complexes coupled to hormones in order to develop new metal-based therapeutic drugs with high selective index for hormone-dependent-breast-cancer treatment (Vera *et al.*, 2011 ▸, 2014 ▸). In connection with the relationship between structure and the activity against hormone-dependent breast cancer, we intend to explore the function­alization of estrogens at C16 position with ferrocene using estrone (**1**) as starting material, due to the versatility which, for synthetic transformations, provides the carbonyl group over other estrogens not containing a carbonyl group. In this context, we present herein the synthesis and crystal structure of 16-ferrocenylmethyl-3β-hy­droxy­estra-1,3,5(10)-trien-17-one dimethyl sulfoxide monosolvate (**2**) and compare it with the structure of estrogen **(1)** and 16β-eth­oxy­methyl­estrone (**3**) (Allan *et al.*, 2006 ▸).
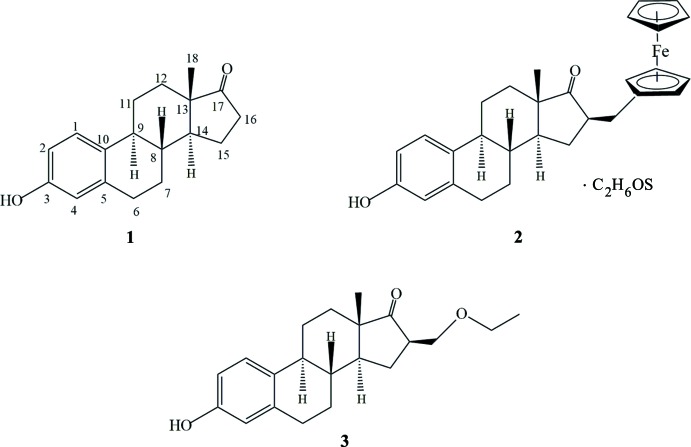


## Structural commentary   

**Figure 1:**
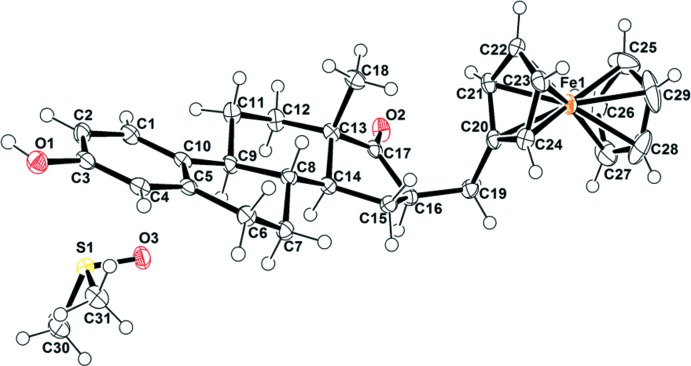
The asymmetric unit of **2**. Displacement ellipsoids are drawn at the 50% probability level.

The ferrocenylmethyl group of **2** is positioned at the beta face of the estrone moiety (Fig. 1 ▸). As a result, a new stereogenic center was formed after substitution at position 16 (C16) of estrone with a ferrocenylmethyl group. This C16 atom has an *R* stereochemical configuration. Table 1 ▸ contains the most relevant bond lengths and angles. The carbonyl bond (C17=O2) of the hormone moiety of **2** is 1.216 (5) Å, which is very similar to in estrogen and 16β-eth­oxy­methyl­estrone [1.215 (2) and 1.219 (2) Å, respectively], corresponding to a carbon–oxygen double (C=O) bond. However, the substitution at C16 of the steroid in **2** and **3**, ferrocenylmethyl and eth­oxy­methyl groups, respectively, makes torsion angles and bond angles at the 16-position slightly different. Both substituents are located on the beta face but, the torsion angle (between C19 and carbonyl group) defined as C19–C16—C17—O2 in **2** is smaller than in **3** (between the carbonyl and the meth­oxy groups), 44.1 (5) and 49.7 (2)°, respectively. The ferrocene moiety is positioned at 112.6 (3)° from C16 (∠C20—C19—C16) while the eth­oxy­methyl group is at 108.4 (1)° (∠C16—C1—O3). The average Fe—C bond length of the substituted Cp ring [Fe—C(Cp*] is similar to the unsubstituted one, 2.048 (3) *vs* 2.040 (12) Å (McAdam *et al.*, 2015 ▸). We might expect that the substitution on the Cp ring with a electron-donating methyl group could enhance the Fe—C(Cp*) bonding, but such an effect is not observed. It is not clear if this is a steric rather than an electronic effect. It is worth mentioning the steroselectivity of this reaction showed the beta steroisomer but it is also the position of the eth­oxy­methyl group on eth­oxy­methyl­estrone. We might expect the beta face of the estrone moiety to be more hindered due to the methyl group on C13 which is located in this face but, according to the mechanism of hydrogen addition to a double bond, the addition is favored on the less hindered alpha face and, as a consequence, the ferrocenyl group is positioned on the beta face.

## Supra­molecular features   

**Figure 2:**
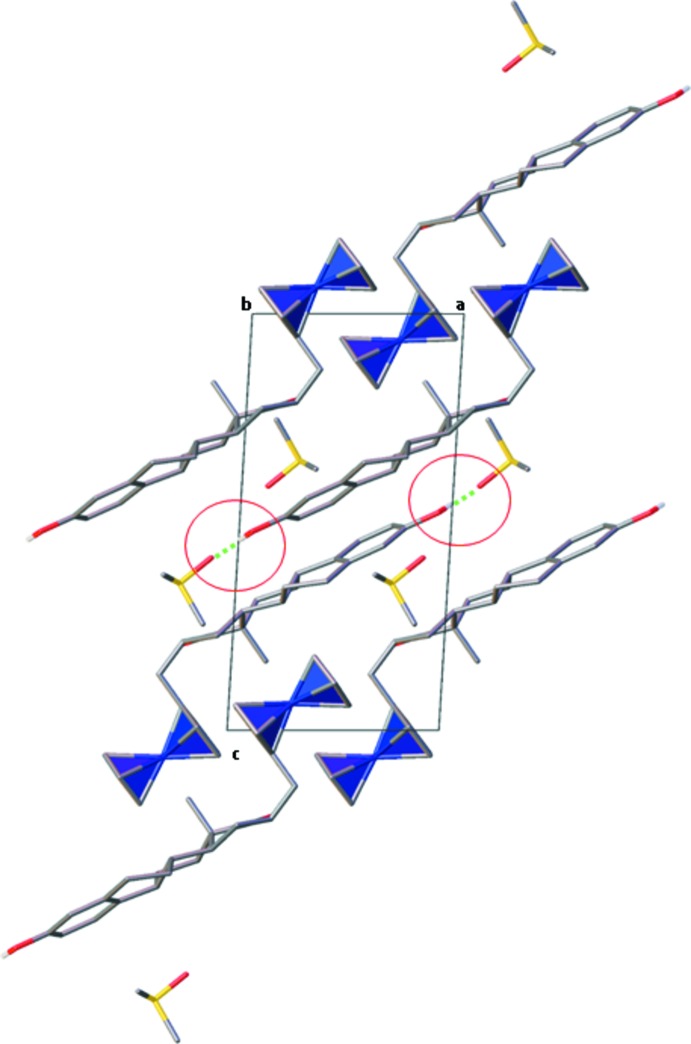
Packing diagram for **2**, projected along the *b* axis. The ferrocene moieties are shown in polyhedral representation for clarity. The O—H⋯O hydrogen bonds are highlighted (in cyan dashed lines).

In the crystal structure of **2** there is a hydrogen bond involving the hydroxyl group at C3 and the DMSO oxygen (Table 2 ▸, Fig. 2 ▸). No head-to-tail hydrogen bonding is observed, as is the case in **1** and **3** (Shikii *et al.*, 2004 ▸; Allan *et al.*, 2006 ▸). In the latter structures, the hydrogen bonds at the two ends are the driving force for packing. It seems that the ferrocenylmethyl substitution on C16 inhibits the hydrogen bonding at the carbonyl oxygen atom, thus eliminating the head-to-tail hydrogen-bonding network existing in **1** and **3**.

## Synthesis and crystallization   

In a 500 mL Parr bottle, 16-ferrocenyl­idene-3β-hy­droxy­estra-1,3,5(10)-trien-17-one complex was dissolved in a mixture of tetra­hydro­furan (THF) and ethanol (1:1) and Pd/C (10wt%, catalytic). The system was purged three times with H_2_ at 40 psi. The reaction mixture was stirred overnight at room temperature under 40 psi of H_2_. The mixture was then filtered through Celite, and the filtrate was evaporated *in vacuo*, resulting in a yellow solid that was purified by column chromatography using CHCl_3_: ethyl acetate (9:1) as mobile phase, affording 67% of **2** as a yellow solid. Yellow rod-shaped crystals were obtained after dissolving the solid 16-ferro­cenyl­methyl-3β-hy­droxy­estra-1,3,5(10)-trien-17-one in a solution of CH_2_Cl_2_ with a few drops of dimethyl sulfoxide, to assure a concentrate solution, layered in hexane.

## Refinement   

Crystal data, data collection and structure refinement details are summarized in Table 3 ▸. H atoms were positioned in idealized locations: *d*(C—H) = 0.95 Å, *U*_iso_(H) = 1.2*U*_eq_(C); *d*(C—H2) = 0.99 Å,*U*_iso_(H) = 1.2 Ueq (C); *d*(C—H3) = 0.98 Å, *U*_iso_(H) = 1.5*U*_eq_(C).

## Supplementary Material

Crystal structure: contains datablock(s) I. DOI: 10.1107/S2056989016008446/bg2586sup1.cif

CCDC reference: 1479699

Additional supporting information:  crystallographic information; 3D view; checkCIF report

## Figures and Tables

**Table 1:** Selected geometrical parameters (Å, °) for compounds **1**, **2** and **3**

	**1**	**2**	**3**
Bond lengths			
Fe—C(Cp)_avg_		2.040 (12)	
Fe—C(Cp*)_subt_		2.048 (3)	
C(Cp)_subt_—CH_2_		1.505 (5)	
C17—O2	1.219 (2)	1.216 (5)	1.215 (2)
C3—O1	1.374 (2)	1.368 (5)	1.371 (2)
			
Hydrogen-bond parameters			
*D*—H	0.86	0.84	0.84
H⋯*A*	1.97 (O2⋯H1)	1.82 [O3(DMSO)⋯H1]	1.93 (O2⋯H1)
*D*⋯*A*	2.819 (2) (O1⋯O2)	2.655 (5) (O1⋯O3)	2.760 (2) (O1⋯O2)
*D*—H⋯*A*	174	174	170
			
Bond angles			
C20—C19—C16		112.6 (3)	
O3—C1—C16			108.4 (1)
			
Torsion angles			
O2—C17—C16—C19		44.1 (5)	49.7 (2) [O(2)—C(17)—C(16)—C(1)]

**Table 2:** Hydrogen-bond geometry (Å, °)

*D*—H⋯*A*	*D*—H	H⋯*A*	*D*⋯*A*	*D*—H⋯*A*
O1—H1⋯O3^i^	0.84	1.82	2.655 (5)	174

Symmetry code: (i) 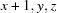.

**Table 3:** Experimental details

Crystal data	
Chemical formula	[Fe(C_5_H_5_)(C_24_H_27_O_2_)]·C_2_H_6_OS
*M*_r_	546.52
Crystal system, space group	Monoclinic, *P*2_1_
Temperature (K)	100
*a*, *b*, *c* (Å)	7.4178 (12), 11.2436 (15), 16.1160 (18)
β (°)	93.148 (4)
*V* (Å^3^)	1342.1 (3)
*Z*	2
Radiation type	Mo *K*α
μ (mm^−1^)	0.67
Crystal size (mm)	0.30 × 0.25 × 0.03

Data collection	
Diffractometer	Bruker APEXII Ultra
Absorption correction	Multi-scan (*SADABS*; Bruker, 2013 ▸)
*T*_min_, *T*_max_	0.064, 0.093
No. of measured, independent and observed [*I* > 2σ(*I*)] reflections	9583, 5327, 4816
*R*_int_	0.048
(sin θ/λ)_max_ (Å^−1^)	0.625

Refinement	
*R*[*F* ^2^ > 2σ(*F* ^2^)], *wR*(*F* ^2^), *S*	0.043, 0.097, 1.02
No. of reflections	5327
No. of parameters	329
No. of restraints	1
H-atom treatment	H-atom parameters constrained
Δρ_max_, Δρ_min_ (e Å^−3^)	0.32, −0.44
Absolute structure	Flack *x* determined using 1990 quotients [(*I* ^+^)−(*I* ^−^)]/[(*I* ^+^)+(*I* ^−^)] (Parsons *et al.*, 2013 ▸)
Absolute structure parameter	0.004 (14)

Computer programs: *APEX2* and *SAINT* (Bruker, 2013 ▸), *SHELXT* (Sheldrick, 2015*a* ▸), *SHELXL2014* (Sheldrick, 2015*b* ▸) and *OLEX2* (Dolomanov *et al.*, 2009 ▸).

## supplementary crystallographic information

### Crystal data

**Table:**

[Fe(C_5_H_5_)(C_24_H_27_O_2_)]·C_2_H_6_OS	*F*(000) = 580
*M**_r_* = 546.52	*D*_x_ = 1.352 Mg m^−^^3^
Monoclinic, *P*2_1_	Mo *K*α radiation, λ = 0.71073 Å
*a* = 7.4178 (12) Å	Cell parameters from 4213 reflections
*b* = 11.2436 (15) Å	θ = 3.0–26.2°
*c* = 16.1160 (18) Å	µ = 0.67 mm^−^^1^
β = 93.148 (4)°	*T* = 100 K
*V* = 1342.1 (3) Å^3^	Block, yellow
*Z* = 2	0.3 × 0.25 × 0.03 mm

### Data collection

**Table:**

Bruker APEXII Ultra diffractometer	5327 independent reflections
Radiation source: Micro Focus Rotating Anode, Bruker TXS	4816 reflections with *I* > 2σ(*I*)
Double Bounce Multilayer Mirrors monochromator	*R*_int_ = 0.048
Detector resolution: 7.9 pixels mm^-1^	θ_max_ = 26.4°, θ_min_ = 2.2°
ω and φ scans	*h* = −9→9
Absorption correction: multi-scan (*SADABS*; Bruker, 2013)	*k* = −13→14
*T*_min_ = 0.064, *T*_max_ = 0.093	*l* = −20→19
9583 measured reflections	

### Refinement

**Table:**

Refinement on *F*^2^	Hydrogen site location: inferred from neighbouring sites
Least-squares matrix: full	H-atom parameters constrained
*R*[*F*^2^ > 2σ(*F*^2^)] = 0.043	*w* = 1/[σ^2^(*F*_o_^2^) + (0.0266*P*)^2^] where *P* = (*F*_o_^2^ + 2*F*_c_^2^)/3
*wR*(*F*^2^) = 0.097	(Δ/σ)_max_ = 0.001
*S* = 1.02	Δρ_max_ = 0.32 e Å^−^^3^
5327 reflections	Δρ_min_ = −0.44 e Å^−^^3^
329 parameters	Absolute structure: Flack *x* determined using 1990 quotients [(*I*^+^)-(*I*^-^)]/[(*I*^+^)+(*I*^-^)] (Parsons *et al.*, 2013)
1 restraint	Absolute structure parameter: 0.004 (14)

### Special details

**Table:**

Geometry. All esds (except the esd in the dihedral angle between two l.s. planes) are estimated using the full covariance matrix. The cell esds are taken into account individually in the estimation of esds in distances, angles and torsion angles; correlations between esds in cell parameters are only used when they are defined by crystal symmetry. An approximate (isotropic) treatment of cell esds is used for estimating esds involving l.s. planes.

### Fractional atomic coordinates and isotropic or equivalent isotropic displacement parameters (Å^2^)

**Table:**

	*x*	*y*	*z*	*U*_iso_*/*U*_eq_	
Fe1	−0.29444 (8)	0.50023 (5)	1.06433 (3)	0.01977 (16)	
S1	0.26603 (16)	0.46255 (9)	0.35262 (6)	0.0224 (3)	
O3	0.1244 (5)	0.4378 (3)	0.41388 (17)	0.0285 (8)	
O2	−0.2299 (4)	0.2860 (3)	0.79220 (17)	0.0247 (7)	
O1	0.9351 (4)	0.6126 (3)	0.47762 (17)	0.0232 (7)	
H1	0.9886	0.5548	0.4569	0.035*	
C17	−0.1502 (6)	0.3780 (4)	0.7790 (2)	0.0176 (9)	
C13	0.0422 (6)	0.3895 (4)	0.7523 (2)	0.0181 (9)	
C8	0.2258 (6)	0.5542 (4)	0.6908 (2)	0.0163 (9)	
H8	0.3108	0.5484	0.7410	0.020*	
C15	−0.0744 (6)	0.5880 (4)	0.7724 (2)	0.0188 (9)	
H15A	−0.1175	0.6629	0.7458	0.023*	
H15B	−0.0055	0.6068	0.8251	0.023*	
C14	0.0394 (5)	0.5159 (4)	0.7143 (2)	0.0170 (8)	
H14	−0.0347	0.5095	0.6608	0.020*	
C22	−0.0406 (7)	0.4352 (4)	1.0540 (3)	0.0219 (10)	
H22	0.0203	0.3809	1.0910	0.026*	
C5	0.5284 (6)	0.6214 (4)	0.5951 (2)	0.0166 (9)	
C10	0.4657 (5)	0.5020 (4)	0.58935 (19)	0.0168 (8)	
C21	−0.1534 (6)	0.4044 (4)	0.9831 (3)	0.0202 (10)	
H21	−0.1811	0.3259	0.9646	0.024*	
C24	−0.1427 (7)	0.6073 (4)	0.9925 (3)	0.0212 (10)	
H24	−0.1619	0.6894	0.9811	0.025*	
C12	0.1022 (6)	0.3007 (4)	0.6877 (2)	0.0225 (10)	
H12A	0.0111	0.2978	0.6405	0.027*	
H12B	0.1112	0.2204	0.7127	0.027*	
C2	0.7268 (6)	0.4550 (4)	0.5087 (2)	0.0198 (9)	
H2	0.7945	0.3980	0.4800	0.024*	
C7	0.2328 (7)	0.6806 (4)	0.6564 (3)	0.0195 (10)	
H7A	0.1600	0.6854	0.6031	0.023*	
H7B	0.1813	0.7368	0.6960	0.023*	
C19	−0.3372 (5)	0.5193 (4)	0.8660 (2)	0.0208 (9)	
H19A	−0.3982	0.5976	0.8641	0.025*	
H19B	−0.4316	0.4571	0.8671	0.025*	
C30	0.1457 (6)	0.4879 (5)	0.2554 (2)	0.0270 (10)	
H30A	0.0824	0.4150	0.2375	0.041*	
H30B	0.2308	0.5106	0.2137	0.041*	
H30C	0.0578	0.5519	0.2615	0.041*	
C1	0.5698 (6)	0.4215 (4)	0.5459 (2)	0.0200 (9)	
H1A	0.5317	0.3410	0.5417	0.024*	
C11	0.2860 (6)	0.3367 (4)	0.6560 (3)	0.0218 (10)	
H11A	0.3801	0.3274	0.7015	0.026*	
H11B	0.3164	0.2827	0.6103	0.026*	
C20	−0.2179 (5)	0.5112 (5)	0.9444 (2)	0.0184 (9)	
C3	0.7837 (6)	0.5728 (4)	0.5137 (2)	0.0192 (10)	
C16	−0.2314 (5)	0.5037 (4)	0.7870 (2)	0.0200 (8)	
H16	−0.3205	0.5140	0.7386	0.024*	
C6	0.4272 (6)	0.7143 (4)	0.6427 (2)	0.0193 (9)	
H6A	0.4283	0.7904	0.6119	0.023*	
H6B	0.4916	0.7272	0.6974	0.023*	
C4	0.6851 (6)	0.6544 (4)	0.5575 (2)	0.0182 (9)	
H4	0.7257	0.7344	0.5619	0.022*	
C29	−0.3916 (9)	0.5424 (7)	1.1756 (3)	0.059 (2)	
H29	−0.3244	0.5803	1.2200	0.071*	
C31	0.3441 (7)	0.6100 (4)	0.3730 (3)	0.0266 (11)	
H31A	0.2407	0.6624	0.3804	0.040*	
H31B	0.4111	0.6383	0.3261	0.040*	
H31C	0.4235	0.6106	0.4236	0.040*	
C18	0.1621 (6)	0.3784 (4)	0.8337 (2)	0.0225 (10)	
H18A	0.1284	0.4401	0.8728	0.034*	
H18B	0.2891	0.3881	0.8214	0.034*	
H18C	0.1445	0.2998	0.8584	0.034*	
C23	−0.0348 (6)	0.5606 (4)	1.0600 (2)	0.0224 (10)	
H23	0.0299	0.6055	1.1018	0.027*	
C28	−0.4933 (9)	0.5984 (6)	1.1132 (4)	0.0490 (17)	
H28	−0.5090	0.6819	1.1079	0.059*	
C9	0.2869 (6)	0.4657 (4)	0.6246 (2)	0.0177 (9)	
H9	0.1941	0.4698	0.5773	0.021*	
C26	−0.5157 (9)	0.4035 (6)	1.0890 (4)	0.0523 (18)	
H26	−0.5483	0.3294	1.0642	0.063*	
C27	−0.5681 (6)	0.5152 (7)	1.0601 (3)	0.0412 (14)	
H27	−0.6432	0.5311	1.0117	0.049*	
C25	−0.4046 (9)	0.4208 (7)	1.1623 (4)	0.059 (2)	
H25	−0.3493	0.3603	1.1960	0.071*	

### Atomic displacement parameters (Å^2^)

**Table:**

	*U*^11^	*U*^22^	*U*^33^	*U*^12^	*U*^13^	*U*^23^
Fe1	0.0179 (3)	0.0243 (3)	0.0174 (3)	−0.0018 (3)	0.0034 (2)	−0.0014 (3)
S1	0.0250 (6)	0.0214 (6)	0.0210 (5)	0.0067 (5)	0.0035 (4)	0.0027 (4)
O3	0.039 (2)	0.0249 (18)	0.0233 (15)	0.0017 (15)	0.0130 (14)	0.0014 (13)
O2	0.0264 (19)	0.0243 (17)	0.0239 (16)	−0.0099 (15)	0.0072 (14)	−0.0039 (13)
O1	0.0227 (18)	0.0230 (17)	0.0243 (16)	−0.0023 (14)	0.0042 (13)	0.0020 (13)
C17	0.019 (2)	0.023 (2)	0.0106 (18)	−0.0015 (19)	−0.0024 (16)	−0.0033 (17)
C13	0.020 (2)	0.018 (2)	0.016 (2)	−0.0036 (19)	0.0002 (17)	−0.0001 (17)
C8	0.019 (2)	0.016 (2)	0.0147 (18)	0.0000 (18)	−0.0002 (16)	−0.0002 (16)
C15	0.020 (2)	0.018 (2)	0.018 (2)	0.0025 (19)	0.0000 (17)	−0.0006 (17)
C14	0.019 (2)	0.018 (2)	0.0145 (17)	−0.0011 (19)	0.0001 (14)	−0.0002 (17)
C22	0.021 (3)	0.025 (3)	0.020 (2)	0.006 (2)	0.0048 (19)	0.0007 (19)
C5	0.019 (2)	0.019 (2)	0.0121 (19)	0.0009 (18)	−0.0024 (16)	0.0005 (16)
C10	0.020 (2)	0.0190 (19)	0.0116 (16)	−0.001 (2)	−0.0015 (14)	0.002 (2)
C21	0.022 (3)	0.023 (2)	0.016 (2)	−0.003 (2)	0.0014 (18)	−0.0052 (18)
C24	0.019 (2)	0.020 (2)	0.024 (2)	−0.003 (2)	0.0042 (19)	−0.0035 (19)
C12	0.026 (3)	0.018 (2)	0.024 (2)	−0.007 (2)	0.0057 (19)	−0.0033 (18)
C2	0.024 (2)	0.019 (2)	0.0165 (19)	0.0005 (19)	0.0031 (17)	−0.0040 (17)
C7	0.024 (3)	0.017 (2)	0.018 (2)	0.0002 (19)	0.0030 (18)	−0.0009 (17)
C19	0.014 (2)	0.027 (3)	0.0211 (18)	0.001 (2)	0.0012 (15)	−0.0017 (19)
C30	0.029 (2)	0.029 (3)	0.0219 (19)	−0.002 (2)	−0.0025 (17)	0.000 (2)
C1	0.025 (2)	0.019 (2)	0.0168 (19)	−0.0036 (19)	0.0014 (17)	0.0016 (17)
C11	0.028 (3)	0.016 (2)	0.022 (2)	−0.005 (2)	0.0076 (19)	−0.0018 (18)
C20	0.0141 (19)	0.025 (2)	0.0164 (17)	−0.001 (2)	0.0029 (14)	−0.0025 (19)
C3	0.018 (2)	0.025 (2)	0.0145 (19)	−0.0017 (19)	−0.0002 (17)	0.0051 (18)
C16	0.017 (2)	0.025 (2)	0.0171 (17)	0.000 (2)	−0.0013 (15)	0.000 (2)
C6	0.026 (2)	0.014 (2)	0.018 (2)	−0.0020 (18)	0.0011 (17)	−0.0023 (17)
C4	0.022 (2)	0.017 (2)	0.0158 (19)	−0.0018 (18)	−0.0022 (17)	0.0018 (16)
C29	0.030 (3)	0.121 (7)	0.029 (3)	−0.024 (4)	0.018 (2)	−0.026 (3)
C31	0.027 (3)	0.028 (3)	0.024 (2)	−0.007 (2)	−0.0020 (19)	0.001 (2)
C18	0.022 (2)	0.024 (2)	0.021 (2)	−0.006 (2)	0.0005 (18)	0.0057 (18)
C23	0.017 (3)	0.033 (3)	0.017 (2)	−0.003 (2)	−0.0016 (18)	−0.003 (2)
C28	0.033 (4)	0.043 (4)	0.074 (4)	0.003 (3)	0.033 (3)	−0.009 (3)
C9	0.024 (2)	0.016 (2)	0.0133 (17)	−0.0004 (18)	0.0009 (16)	−0.0023 (16)
C26	0.040 (4)	0.053 (4)	0.067 (4)	−0.028 (3)	0.030 (3)	−0.022 (3)
C27	0.018 (2)	0.076 (5)	0.030 (2)	0.004 (3)	0.0067 (19)	0.006 (3)
C25	0.039 (4)	0.092 (6)	0.049 (4)	0.016 (4)	0.020 (3)	0.048 (4)

### Geometric parameters (Å, º)

**Table:**

Fe1—C22	2.035 (5)	C12—H12A	0.9900
Fe1—C21	2.030 (4)	C12—H12B	0.9900
Fe1—C24	2.049 (5)	C12—C11	1.536 (6)
Fe1—C20	2.047 (3)	C2—H2	0.9500
Fe1—C29	2.026 (5)	C2—C1	1.391 (6)
Fe1—C23	2.047 (5)	C2—C3	1.392 (6)
Fe1—C28	2.035 (6)	C7—H7A	0.9900
Fe1—C26	2.026 (6)	C7—H7B	0.9900
Fe1—C27	2.035 (5)	C7—C6	1.518 (6)
Fe1—C25	2.025 (6)	C19—H19A	0.9900
S1—O3	1.506 (3)	C19—H19B	0.9900
S1—C30	1.783 (4)	C19—C20	1.505 (5)
S1—C31	1.781 (4)	C19—C16	1.543 (5)
O2—C17	1.216 (5)	C30—H30A	0.9800
O1—H1	0.8400	C30—H30B	0.9800
O1—C3	1.368 (5)	C30—H30C	0.9800
C17—C13	1.518 (6)	C1—H1A	0.9500
C17—C16	1.545 (6)	C11—H11A	0.9900
C13—C14	1.548 (6)	C11—H11B	0.9900
C13—C12	1.527 (5)	C11—C9	1.536 (6)
C13—C18	1.548 (5)	C3—C4	1.389 (6)
C8—H8	1.0000	C16—H16	1.0000
C8—C14	1.516 (5)	C6—H6A	0.9900
C8—C7	1.527 (6)	C6—H6B	0.9900
C8—C9	1.545 (5)	C4—H4	0.9500
C15—H15A	0.9900	C29—H29	0.9500
C15—H15B	0.9900	C29—C28	1.376 (9)
C15—C14	1.527 (5)	C29—C25	1.387 (10)
C15—C16	1.529 (6)	C31—H31A	0.9800
C14—H14	1.0000	C31—H31B	0.9800
C22—H22	0.9500	C31—H31C	0.9800
C22—C21	1.422 (6)	C18—H18A	0.9800
C22—C23	1.414 (6)	C18—H18B	0.9800
C5—C10	1.422 (6)	C18—H18C	0.9800
C5—C6	1.519 (6)	C23—H23	0.9500
C5—C4	1.390 (6)	C28—H28	0.9500
C10—C1	1.402 (6)	C28—C27	1.365 (9)
C10—C9	1.527 (5)	C9—H9	1.0000
C21—H21	0.9500	C26—H26	0.9500
C21—C20	1.423 (7)	C26—C27	1.387 (10)
C24—H24	0.9500	C26—C25	1.416 (9)
C24—C20	1.425 (6)	C27—H27	0.9500
C24—C23	1.415 (6)	C25—H25	0.9500
			
C22—Fe1—C24	68.00 (19)	C1—C2—C3	119.4 (4)
C22—Fe1—C20	68.81 (16)	C3—C2—H2	120.3
C22—Fe1—C23	40.53 (16)	C8—C7—H7A	109.7
C22—Fe1—C28	156.9 (2)	C8—C7—H7B	109.7
C21—Fe1—C22	40.95 (17)	H7A—C7—H7B	108.2
C21—Fe1—C24	68.24 (16)	C6—C7—C8	109.7 (4)
C21—Fe1—C20	40.86 (19)	C6—C7—H7A	109.7
C21—Fe1—C23	68.60 (19)	C6—C7—H7B	109.7
C21—Fe1—C28	161.4 (2)	H19A—C19—H19B	107.8
C21—Fe1—C27	124.8 (2)	C20—C19—H19A	109.1
C20—Fe1—C24	40.73 (18)	C20—C19—H19B	109.1
C29—Fe1—C22	122.2 (2)	C20—C19—C16	112.6 (3)
C29—Fe1—C21	156.4 (3)	C16—C19—H19A	109.1
C29—Fe1—C24	126.6 (2)	C16—C19—H19B	109.1
C29—Fe1—C20	162.2 (3)	S1—C30—H30A	109.5
C29—Fe1—C23	109.5 (2)	S1—C30—H30B	109.5
C29—Fe1—C28	39.6 (3)	S1—C30—H30C	109.5
C29—Fe1—C26	67.7 (3)	H30A—C30—H30B	109.5
C29—Fe1—C27	66.9 (2)	H30A—C30—H30C	109.5
C29—Fe1—C25	40.0 (3)	H30B—C30—H30C	109.5
C23—Fe1—C24	40.42 (18)	C10—C1—H1A	118.7
C23—Fe1—C20	68.75 (16)	C2—C1—C10	122.5 (4)
C28—Fe1—C24	109.4 (2)	C2—C1—H1A	118.7
C28—Fe1—C20	125.3 (2)	C12—C11—H11A	109.2
C28—Fe1—C23	122.5 (2)	C12—C11—H11B	109.2
C26—Fe1—C22	125.9 (3)	H11A—C11—H11B	107.9
C26—Fe1—C21	107.1 (2)	C9—C11—C12	112.2 (4)
C26—Fe1—C24	154.4 (2)	C9—C11—H11A	109.2
C26—Fe1—C20	119.2 (2)	C9—C11—H11B	109.2
C26—Fe1—C23	163.4 (3)	C21—C20—Fe1	68.9 (2)
C26—Fe1—C28	66.6 (2)	C21—C20—C24	106.9 (3)
C26—Fe1—C27	39.9 (3)	C21—C20—C19	125.9 (4)
C27—Fe1—C22	162.4 (2)	C24—C20—Fe1	69.7 (2)
C27—Fe1—C24	120.7 (2)	C24—C20—C19	127.3 (5)
C27—Fe1—C20	106.95 (17)	C19—C20—Fe1	128.0 (3)
C27—Fe1—C23	155.6 (2)	O1—C3—C2	122.7 (4)
C27—Fe1—C28	39.2 (3)	O1—C3—C4	117.9 (4)
C25—Fe1—C22	108.7 (2)	C4—C3—C2	119.4 (4)
C25—Fe1—C21	120.9 (3)	C17—C16—H16	106.6
C25—Fe1—C24	163.2 (3)	C15—C16—C17	104.5 (3)
C25—Fe1—C20	155.1 (3)	C15—C16—C19	118.9 (3)
C25—Fe1—C23	126.4 (2)	C15—C16—H16	106.6
C25—Fe1—C28	66.7 (3)	C19—C16—C17	113.0 (4)
C25—Fe1—C26	40.9 (3)	C19—C16—H16	106.6
C25—Fe1—C27	67.5 (2)	C5—C6—C7	113.7 (3)
O3—S1—C30	105.8 (2)	C5—C6—H6A	108.8
O3—S1—C31	106.4 (2)	C5—C6—H6B	108.8
C31—S1—C30	98.9 (2)	C7—C6—H6A	108.8
C3—O1—H1	109.5	C7—C6—H6B	108.8
O2—C17—C13	126.6 (4)	H6A—C6—H6B	107.7
O2—C17—C16	124.6 (4)	C5—C4—H4	119.3
C13—C17—C16	108.8 (4)	C3—C4—C5	121.4 (4)
C17—C13—C14	101.4 (3)	C3—C4—H4	119.3
C17—C13—C12	116.8 (3)	Fe1—C29—H29	125.0
C17—C13—C18	105.0 (3)	C28—C29—Fe1	70.6 (3)
C14—C13—C18	113.8 (3)	C28—C29—H29	126.1
C12—C13—C14	109.2 (3)	C28—C29—C25	107.9 (6)
C12—C13—C18	110.5 (4)	C25—C29—Fe1	69.9 (4)
C14—C8—H8	108.7	C25—C29—H29	126.1
C14—C8—C7	113.9 (4)	S1—C31—H31A	109.5
C14—C8—C9	107.3 (3)	S1—C31—H31B	109.5
C7—C8—H8	108.7	S1—C31—H31C	109.5
C7—C8—C9	109.4 (3)	H31A—C31—H31B	109.5
C9—C8—H8	108.7	H31A—C31—H31C	109.5
H15A—C15—H15B	109.2	H31B—C31—H31C	109.5
C14—C15—H15A	111.3	C13—C18—H18A	109.5
C14—C15—H15B	111.3	C13—C18—H18B	109.5
C14—C15—C16	102.5 (3)	C13—C18—H18C	109.5
C16—C15—H15A	111.3	H18A—C18—H18B	109.5
C16—C15—H15B	111.3	H18A—C18—H18C	109.5
C13—C14—H14	105.7	H18B—C18—H18C	109.5
C8—C14—C13	111.5 (3)	Fe1—C23—H23	126.3
C8—C14—C15	123.1 (4)	C22—C23—Fe1	69.3 (3)
C8—C14—H14	105.7	C22—C23—C24	107.6 (4)
C15—C14—C13	103.9 (3)	C22—C23—H23	126.2
C15—C14—H14	105.7	C24—C23—Fe1	69.9 (3)
Fe1—C22—H22	126.2	C24—C23—H23	126.2
C21—C22—Fe1	69.3 (3)	Fe1—C28—H28	126.1
C21—C22—H22	125.9	C29—C28—Fe1	69.8 (4)
C23—C22—Fe1	70.2 (3)	C29—C28—H28	125.3
C23—C22—H22	125.9	C27—C28—Fe1	70.4 (3)
C23—C22—C21	108.2 (4)	C27—C28—C29	109.4 (6)
C10—C5—C6	120.9 (4)	C27—C28—H28	125.3
C4—C5—C10	120.1 (4)	C8—C9—H9	106.3
C4—C5—C6	119.0 (4)	C10—C9—C8	112.2 (3)
C5—C10—C9	121.1 (4)	C10—C9—C11	113.3 (4)
C1—C10—C5	117.1 (4)	C10—C9—H9	106.3
C1—C10—C9	121.7 (4)	C11—C9—C8	111.9 (3)
Fe1—C21—H21	125.8	C11—C9—H9	106.3
C22—C21—Fe1	69.7 (3)	Fe1—C26—H26	125.4
C22—C21—H21	125.8	C27—C26—Fe1	70.3 (3)
C22—C21—C20	108.3 (4)	C27—C26—H26	126.4
C20—C21—Fe1	70.2 (2)	C27—C26—C25	107.2 (6)
C20—C21—H21	125.8	C25—C26—Fe1	69.5 (4)
Fe1—C24—H24	126.8	C25—C26—H26	126.4
C20—C24—Fe1	69.6 (2)	Fe1—C27—H27	125.6
C20—C24—H24	125.5	C28—C27—Fe1	70.4 (3)
C23—C24—Fe1	69.7 (3)	C28—C27—C26	108.2 (5)
C23—C24—H24	125.5	C28—C27—H27	125.9
C23—C24—C20	108.9 (4)	C26—C27—Fe1	69.7 (3)
C13—C12—H12A	109.5	C26—C27—H27	125.9
C13—C12—H12B	109.5	Fe1—C25—H25	125.6
C13—C12—C11	110.6 (3)	C29—C25—Fe1	70.0 (4)
H12A—C12—H12B	108.1	C29—C25—C26	107.3 (6)
C11—C12—H12A	109.5	C29—C25—H25	126.4
C11—C12—H12B	109.5	C26—C25—Fe1	69.6 (3)
C1—C2—H2	120.3	C26—C25—H25	126.4
			
Fe1—C22—C21—C20	59.8 (3)	C7—C8—C9—C10	50.4 (4)
Fe1—C22—C23—C24	−59.6 (3)	C7—C8—C9—C11	179.0 (4)
Fe1—C21—C20—C24	59.6 (3)	C1—C10—C9—C8	162.9 (3)
Fe1—C21—C20—C19	−122.3 (4)	C1—C10—C9—C11	35.0 (5)
Fe1—C24—C20—C21	−59.1 (3)	C1—C2—C3—O1	−179.0 (4)
Fe1—C24—C20—C19	122.9 (4)	C1—C2—C3—C4	1.5 (6)
Fe1—C24—C23—C22	59.2 (4)	C20—C24—C23—Fe1	−58.6 (3)
Fe1—C29—C28—C27	59.4 (4)	C20—C24—C23—C22	0.5 (6)
Fe1—C29—C25—C26	−59.9 (4)	C20—C19—C16—C17	65.7 (5)
Fe1—C28—C27—C26	59.7 (4)	C20—C19—C16—C15	−57.3 (6)
Fe1—C26—C27—C28	−60.1 (4)	C3—C2—C1—C10	−0.4 (6)
Fe1—C26—C25—C29	60.2 (5)	C16—C17—C13—C14	−20.6 (4)
O2—C17—C13—C14	159.3 (4)	C16—C17—C13—C12	−139.1 (3)
O2—C17—C13—C12	40.7 (6)	C16—C17—C13—C18	98.1 (4)
O2—C17—C13—C18	−82.0 (5)	C16—C15—C14—C13	−43.4 (4)
O2—C17—C16—C15	174.7 (4)	C16—C15—C14—C8	−171.2 (3)
O2—C17—C16—C19	44.1 (5)	C16—C19—C20—Fe1	−169.8 (4)
O1—C3—C4—C5	179.2 (4)	C16—C19—C20—C21	−79.7 (5)
C17—C13—C14—C8	173.9 (3)	C16—C19—C20—C24	97.9 (5)
C17—C13—C14—C15	39.3 (4)	C6—C5—C10—C1	−178.5 (3)
C17—C13—C12—C11	170.1 (4)	C6—C5—C10—C9	5.1 (5)
C13—C17—C16—C15	−5.4 (4)	C6—C5—C4—C3	179.5 (3)
C13—C17—C16—C19	−136.0 (3)	C4—C5—C10—C1	1.0 (5)
C13—C12—C11—C9	−53.2 (5)	C4—C5—C10—C9	−175.5 (3)
C8—C7—C6—C5	49.2 (4)	C4—C5—C6—C7	161.2 (4)
C14—C13—C12—C11	55.8 (5)	C29—C28—C27—Fe1	−59.1 (4)
C14—C8—C7—C6	174.5 (3)	C29—C28—C27—C26	0.6 (6)
C14—C8—C9—C10	174.3 (3)	C18—C13—C14—C8	61.8 (4)
C14—C8—C9—C11	−57.1 (4)	C18—C13—C14—C15	−72.8 (4)
C14—C15—C16—C17	29.6 (4)	C18—C13—C12—C11	−70.0 (4)
C14—C15—C16—C19	156.7 (3)	C23—C22—C21—Fe1	−59.6 (4)
C22—C21—C20—Fe1	−59.5 (3)	C23—C22—C21—C20	0.2 (6)
C22—C21—C20—C24	0.1 (4)	C23—C24—C20—Fe1	58.7 (3)
C22—C21—C20—C19	178.1 (4)	C23—C24—C20—C21	−0.4 (5)
C5—C10—C1—C2	−0.8 (6)	C23—C24—C20—C19	−178.4 (4)
C5—C10—C9—C8	−20.9 (5)	C28—C29—C25—Fe1	60.7 (4)
C5—C10—C9—C11	−148.8 (3)	C28—C29—C25—C26	0.8 (8)
C10—C5—C6—C7	−19.3 (5)	C9—C8—C14—C13	61.4 (4)
C10—C5—C4—C3	0.0 (6)	C9—C8—C14—C15	−174.1 (3)
C21—C22—C23—Fe1	59.1 (3)	C9—C8—C7—C6	−65.6 (4)
C21—C22—C23—C24	−0.4 (6)	C9—C10—C1—C2	175.6 (4)
C12—C13—C14—C8	−62.1 (4)	C27—C26—C25—Fe1	−60.5 (4)
C12—C13—C14—C15	163.2 (3)	C27—C26—C25—C29	−0.4 (7)
C12—C11—C9—C8	54.3 (5)	C25—C29—C28—Fe1	−60.3 (5)
C12—C11—C9—C10	−177.6 (3)	C25—C29—C28—C27	−0.9 (7)
C2—C3—C4—C5	−1.3 (6)	C25—C26—C27—Fe1	60.0 (4)
C7—C8—C14—C13	−177.5 (3)	C25—C26—C27—C28	−0.2 (6)
C7—C8—C14—C15	−53.0 (5)		

### Hydrogen-bond geometry (Å, º)

**Table:**

*D*—H···*A*	*D*—H	H···*A*	*D*···*A*	*D*—H···*A*
O1—H1···O3^i^	0.84	1.82	2.655 (5)	174

Symmetry code: (i) *x*+1, *y*, *z*.
